# Trichostatin A promotes esophageal squamous cell carcinoma cell migration and EMT through BRD4/ERK1/2‐dependent pathway

**DOI:** 10.1002/cam4.4059

**Published:** 2021-06-23

**Authors:** Danhui Liu, Yuzhen Liu, Bo Qi, Chengwei Gu, Shuhua Huo, Baosheng Zhao

**Affiliations:** ^1^ Department of Thoracic Surgery The First Affiliated Hospital of Xinxiang Medical University Weihui China; ^2^ Esophageal Cancer Institute of Xinxiang Medical University Weihui China; ^3^ Life Science Research Center The First Affiliated Hospital of Xinxiang Medical University Weihui China

**Keywords:** EMT, esophageal squamous cell carcinoma, histone deacetylase inhibitor, migration, trichostatin A

## Abstract

**Background:**

Histone deacetylases (HDACs) have been demonstrated to be aberrantly activated in tumorigenesis and cancer development. Thus, HDAC inhibitors (HDACIs) are considered to be promising anti‐cancer therapeutics. However, recent studies have shown that HDACIs promote the migration of many cancer cells. Therefore, there is a need to elucidate the underlying mechanisms of HDACIs on cancer cell migration to establish a combination therapy that overcomes HDACI‐induced cell migration.

**Methods:**

KYSE‐150 and EC9706 cells were treated differently. Effects of drugs and siRNA treatment on tumor cell migration and cell signaling pathways were investigated by transwell migration assy. Gene expression for SNAI2 was tested by RT‐qPCR. Western blot analysis was employed to detect the level of E‐cadherin, β‐catenin, vimentin,Slug，ERK1/2, H3, PAI‐1 and BRD4. The effect of drugs on cell morphology was evaluated through phase‐contrast microscopic images.

**Results:**

TSA promotes epithelial‐mesenchymal transition (EMT) in ESCC cells by downregulating the epithelial marker E‐cadherin and upregulating mesenchymal markers β‐catenin, vimentin, Slug, and PAI‐1. Knockdown of Slug by siRNA or inhibition of PAI‐1 clearly suppressed TSA‐induced ESCC cell migration and resulted in the reversal of TSA‐triggered E‐cadherin, β‐catenin, and vimentin expression. However, no crosstalk between Slug and PAI‐1 was observed in TSA‐treated ESCC cells. Blocking ERK1/2 activation also inhibited TSA‐induced ESCC cell migration, EMT, and upregulation of Slug and PAI‐1 levels in ESCC cells. Interestingly, inhibition of BRD4 suppressed TSA‐induced ESCC cell migration and attenuated TSA‐induced ERK1/2 activation and upregulation of Slug and PAI‐1 levels.

**Conclusions:**

Our data indicate the existence of at least two separable ERK1/2‐dependent signaling pathways in TSA‐mediated ESCC cell migration: an ERK1/2–Slug branch and an ERK1/2‐PAI‐1 branch. Both branches of TSA‐induced ESCC cell migration appear to favor the EMT process, while BRD4 is responsible for two separable ERK1/2‐dependent signaling pathways in TSA‐mediated ESCC cell migration.

## Graphical abstract

Histone deacetylases (HDACs) have been demonstrated to be aberrantly activated in tumorigenesis and cancer development, thus HDAC inhibitors (HDACIs) are considered to be promising anti‐cancer therapeutics. However, recent studies have shown that HDACIs promote the migration of multiple cancer cells. This study clarifies the mechanism of TSA promoting the migration of esophageal squamous cell carcinoma cells.

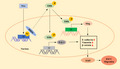

## 1 INTRODUCTION

Esophageal cancer is a common malignant tumor of the digestive system and is mainly divided into two major histopathological types: esophageal squamous cell carcinoma (ESCC) and esophageal adenocarcinoma.^1^ More than half of the global patients with esophageal cancer are Chinese.^2^ Affected by factors, such as diet and genetics, more than 97% of esophageal cancers are ESCC in China.^3^ Various therapeutic approaches are currently available for the treatment of ESCC. However, the outcomes of ESCC patients remain poor and the 5‐year survival rate is still lower than 20% in China.^4^ Therefore, there is an urgent need to develop alternative and novel approaches that could effectively prevent ESCC cell growth and metastasis to improve ESCC patient prognosis.

Histone acetylation, dynamically controlled by histone acetylases (HATs) and histone deacetylases (HDACs), is a major type of epigenetic modification that plays a critical role in a number of biological processes by regulating chromatin structure and gene transcription. Mutations and/or abnormal expression of numerous HDACs have been demonstrated in cancer,^5^, ^6^ and deregulation of the global pattern of histone acetylation has been associated with tumorigenesis and development.^7^, ^8^ Therefore, HDAC inhibitors (HDACIs) are considered to be promising therapeutic agents against human cancers.^9^, ^10^, ^11^, ^12^ Inhibition of the activity of HDAC by HDACIs results in the maintenance of the high acetylation state of histones and non‐histone proteins, subsequently leading to the modification and alteration of transcription factor complexes.^13^, ^14^ Multiple mechanisms, including the promotion of cancer cell apoptosis and cell‐cycle arrest, are implicated in the anti‐cancer actions of HDACIs. As a result, several HDACIs, such as suberoylanilide hydroxamic acid (SAHA), have been used clinically to treat hematological malignancies.^15^ Despite these advancements, the outcomes of patients with solid tumors are poor in HDACI clinical trials. In recent years, studies have shown that HDACIs promote the migration of many cancer cells derived from liver, colon, and lung by activating tumor‐promoting genes.^16^, ^17^, ^18^ Thus, there is a need to elucidate the mechanisms by which HDACIs exert anti‐cancer activity in many cancer cell types. Drug combinations that target HDACI‐activated migration can provide improved therapeutic benefits in treating cancer patients with HDACIs.

HDAC1 mRNA has been reported to be elevated in ESCC samples, and HDAC2 levels have been correlated with the invasion depth of ESCC.^19^, ^20^ Related research has reported that trichostatin A (TSA), a broad‐spectrum HDACI, suppresses proliferation and promotes apoptosis of ESCC cells.^21^, ^22^ Despite these reports, there is little literature on the effect of HDACIs on ESCC cell migration.

Previous study showed that TSA promoted the migration of ESCC cells.^23^ In this study, the mechanism by which TSA promotes ESCC cell migration has been explored. We found that TSA enhanced epithelial‐mesenchymal transition (EMT) through the upregulation of the transcription factor Slug and mesenchymal markers in ESCC cells. Our research provides insights into the unsatisfactory effect of HDACIs on solid tumors and a reasonable guide for the use of HDACIs in anti‐cancer clinical treatment.

## 2 MATERIALS AND METHODS

### 2.1 Cells and cell culture

The human esophageal squamous cell carcinoma cell lines KYSE‐150 and EC9706 were purchased from the Cell Bank of the Typical Culture Preservation Committee of the Chinese Academy of Sciences. The two cell lines were cultured in RPMI 1640 medium (Corning) containing 10% fetal bovine serum (Gibco).

### 2.2 Transwell migration assay

The migration of the cells was studied using a 6.5 mm‐diameter transwell chamber insert with 8‐μm pore size (Corning). Two hundred microliters of cell suspension (KYSE‐150 5.0 × 10^5^ cells/ml, EC9706 3.0 × 10^5^ cells/ml) were seeded into the upper chamber with or without application of drugs, which were as follows: TSA (Beyotime Biotechnology), U0126‐EtOH, PD98059, PAI‐039, and JQ1 (APExBIO). The lower chambers contained 600 μl of RPMI 1640 medium supplemented with 10% fetal bovine serum. After incubation for 24 h, the cells that migrated to the lower surface of the membrane of the upper chamber were fixed with 4% paraformaldehyde and stained with crystal violet. The non‐migrating cells in the upper surface of the membrane were removed. Five random fields of each membrane were captured using a phase‐contrast microscope (Nikon), and the cells that migrated were counted.

### 2.3 Cell morphological observation

KYSE‐150 and EC9706 cells were seeded in six‐well plates (KYS‐150 2.5 × 10^5^ cells/well, EC9706 3.5 × 10^5^ cells/well). After overnight incubation, cells were treated with or without different inhibitors for 24 h. Five different fields were selected to observe cell phenotypic changes using a phase‐contrast microscope (Nikon).

### 2.4 Reverse transcription real‐time quantitative polymerase chain reaction (RT‐qPCR)

RNA was extracted with TRIzol (Invitrogen) and 1 µg of RNA was transcribed into cDNA using the QuantiNova^®^ Reverse Transcription Kit (Qiagen). PCR was conducted with 2 μl of cDNA as the template using QuantiNova^®^ SYBR^®^ Green PCR Kit (Qiagen) following protocol: 95°C for 30 s, 40 cycles consisting of 95°C for 5 s and 60°C for 34 s. Primers were ordered from BGI and the sequences were as follows: SNAI2 forward: 5′‐AGCCAAACTACAGCGAACTG‐3′ and reverse: 5′‐GGTCTGAAAGCTTGGACTGT‐3′; and GAPDH forward: 5′‐GCAGGGGGGAGCCAAAAGGGT‐3′ and reverse: 5′‐TGGGTGGCAGTATGGCTCG‐3′. GAPDH was used as an internal control. The relative expression of SNAI2 RNA was calculated using the comparative cycle threshold and the 2^−ΔΔ^Ct method.

### 2.5 Small interfering RNA (siRNA) transfection

KYSE‐150 (2.5 × 10^5^) and EC9706 (3.5 × 10^5^) cells were cultured in six‐well plates overnight and siRNA was transfected into the cells using Lipofectamine 2000 reagent (Invitrogen) according to the manufacturer's instructions. The sequences of siRNA (GenePharma) were as follows: SNAI2 forward: 5′‐CCGUAUCUCUAUGAGUUACUCCA‐3′ and reverse: 5′‐UGGAGUAACUCUCAUAGAGAUACGG‐3′; and negative control forward: 5′‐UUCUCCGAACGUAGCUTT‐3′ and reverse: 5′‐ACGUGACGUUCGGAGAATT‐3′.

### 2.6 Western blot

KYSE‐150 and EC9706 cells were lysed in RIPA buffer. Protein concentration was determined using BCA Protein Assay Kit (Beyotime Biotechnology). Thirty micrograms of protein from each group were electrophoresed on 10% SDS‐PAGE and then transferred to a polyvinylidene fluoride membrane (Millipore). The membrane was blocked with 5% nonfat milk (Beyotime Biotechnology) and then individually incubated with the listed primary antibodies (all in a 1:1000 dilution), E‐cadherin, β‐catenin, vimentin, Slug, ERK1/2, p‐ERK1/2, PAI‐1, H3, H3K9Ac, BRD4 (all from Cell Signaling Technology), and GAPDH (Boster). After washing in TBS‐T, the membranes were incubated with HRP‐conjugated secondary antibodies (1:8000, Dingguo) 1 h. After washing, the blots were processed with BeyoECL chemiluminescence kit (Beyotime Biotechnology) and imaged with an Amersham Imager 600 system (GE Healthcare Biosciences).

### 2.7 Statistical analysis

All values are expressed as mean ± standard deviation (SD). The significance of differences between the two groups was tested by Student's *t*‐test and differences were considered significant at *p *< 0.05.

## 3 RESULTS

### 3.1 TSA facilitates ESCC cell migration via regulation of EMT

**FIGURE 1:**
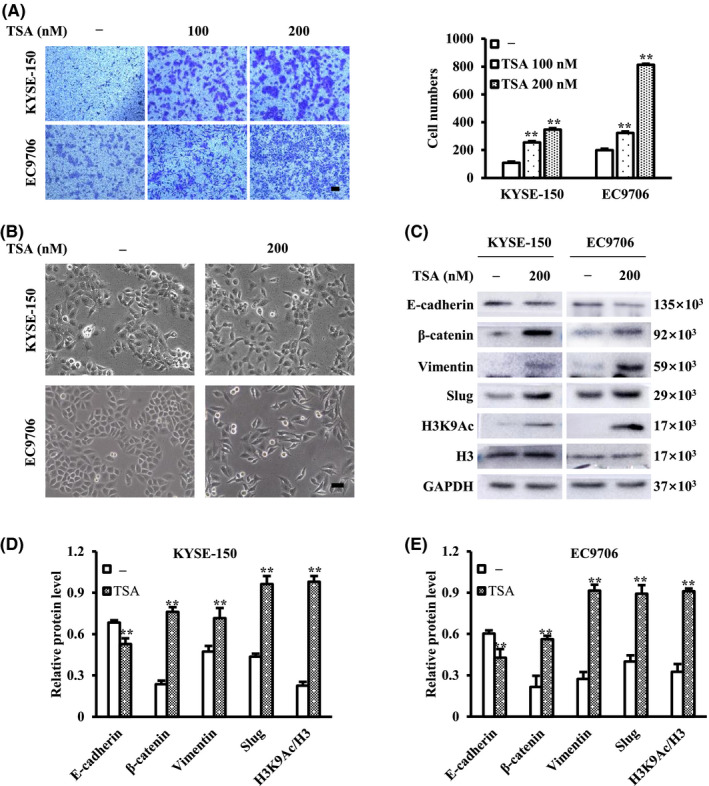
TSA promotes esophageal squamous cell carcinoma (ESCC) cell migration and epithelial‐mesenchymal transition (EMT). (A) TSA‐induced ESCC cell migration was assessed by transwell assay. (B) Phase contrast images of ESCC cells treated with TSA were captured by a Nikon digital microscope. (C–E) Protein expression levels of EMT markers and H3K9Ac were analyzed by western blot. **p < 0.01 versus control. Scale bars in (A) and (B) are 100 μm

Using transwell migration assay, it was found that ESCC cell migration was enhanced after treatment with TSA for 24 h in a dose‐dependent manner (Figure 1A). Compared with the control, TSA‐treated KYSE‐150 and EC9706 cells had stretched and had an elongated spindle‐like phenotype, which is a separable feature of the mesenchymal cells (Figure 1B). TSA not only decreased the expression of E‐cadherin, but also increased the levels of β‐catenin, vimentin, Slug, and H3K9Ac (Figure 1C–1E). These data indicate that TSA facilitates ESCC cell migration by targeting the EMT pathway.

### 3.2 TSA promotes ESCC cell migration through Slug‐mediated EMT

**FIGURE 2:**
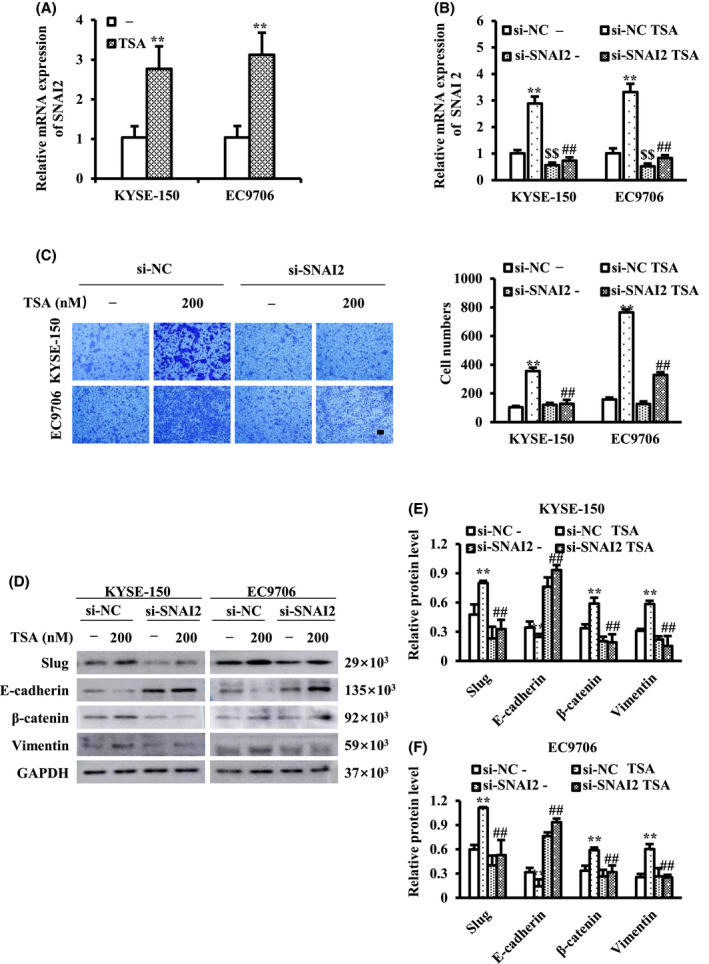
TSA facilitates migration of esophageal squamous cell carcinoma (ESCC) cells by increasing the expression of Slug. (A) Relative levels of SNAI2 mRNA in ESCC cells after treatment with TSA was examined by RT‐qPCR. (B) Relative level of SNAI2 mRNA in control cells and SNAI2‐knockdown cells treated with TSA was examined by RTqPCR. (C) Cell migration of control cells and SNAI2‐knockdown cells was examined by transwell assay, and migrated cells were quantified. (D–F) The expression levels of epithelialmesenchymal transition (EMT) markers in control cells and SNAI2‐knockdown cells after treatment with TSA. Scale bar represents 100 μm. ^**^ *p *< 0.01 versus control, ^$$^ *p *< 0.01 versus control, ^##^ *p *< 0.01 versus TSA

Slug is a transcription factor that is considered to be an important regulator of EMT‐related signaling molecules (SNAI2 is the gene name and Slug is the protein name). To explore whether TSA promotes EMT in ESCC cells by influencing Slug expression, we measured the RNA level of SNAI2 (Figure 2A) and knocked down SNAI2 by the transfection of si‐SNAI2 in ESCC cells. Knockdown of SNAI2, confirmed by RT‐qPCR (Figure 2B), significantly reversed the effect of TSA on ESCC cell migration (Figure 2C). Moreover, knockdown of SNAI2 impaired TSA‐induced downregulation of E‐cadherin and TSA‐induced upregulation of β‐catenin and vimentin (Figure 2D–2F). These results indicate that TSA facilitates ESCC cell migration via slug‐mediated induction of EMT.

### 3.3 TSA promotes ESCC cell migration by activating the ERK1/2‐Slug signaling pathway

**FIGURE 3:**
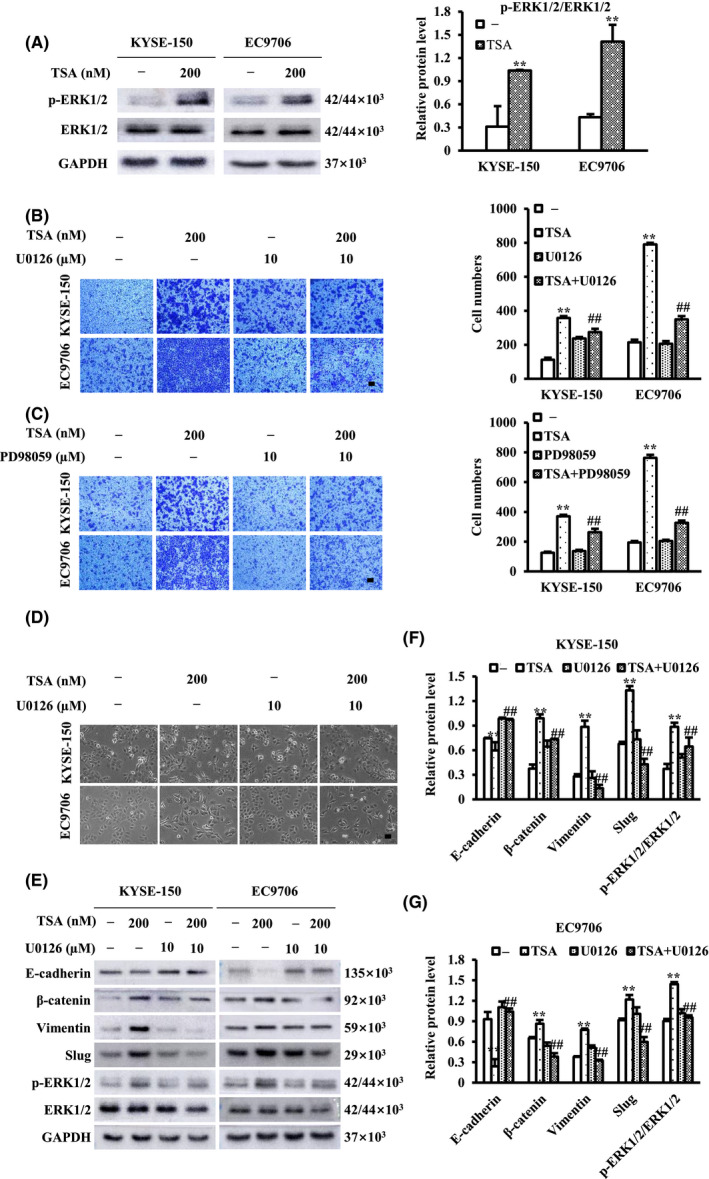
TSA promotes esophageal squamous cell carcinoma (ESCC) cell migration and epithelial‐mesenchymal transition (EMT) by activating ERK1/2 pathway. (A)Phosphorylation of ERK1/2 (p‐ERK1/2) after treatment of ESCC cells with TSA for 24 h was examined by western blot. (B and C) ESCC cells were treated with TSA in the absence or presence of U0126 and PD98059 for 24 h. Cell migration was measured and migrated cells were quantified. (D and E) ESCC cells were treated with 200 nM TSA in the absence or presence of 10 μM U0126. The cell morphology was captured by a phase contrast Nikon microscope equipped with a digital camera (D) and cell lysates were subjected to immunoblot analysis with the appropriate antibodies (E–G). Scale bars represents 100 μm. ^**^ *p *< 0.01 versus control. ^##^ *p *< 0.01 versus TSA

TSA has been demonstrated to activate multiple intracellular signaling pathways, such as ERK1/2.^24^ To investigate the mechanism underlying TSA facilitation of ESCC cell migration, the levels of phosphorylated ERK1/2 (p‐ERK1/2) and the active form of ERK1/2, were examined in TSA‐treated ESCC cells. As shown in Figure 3A, TSA clearly increased the phosphorylation of ERK1/2. Next, we investigated whether TSA regulates ESCC cell migration via the ERK1/2 pathway. Using MEK1/2 inhibitors (U0126 and PD98059), which inhibit ERK1/2 activity, we examined the effect of ERK1/2 on TSA‐induced ESCC cell migration. TSA‐treated ESCC cells were cultured in the absence or presence of U0126 or PD98059, followed by transwell assays. The results revealed that both U0126 and PD98059 prevented TSA‐induced ESCC migration (Figure 3B and 3C). The effect of U0126 on TSA‐induced changes in the morphology of ESCC cells and the levels of EMT markers were also evaluated. Treatment of TSA‐treated cells with U0126 resulted in their transformation from a mesenchymal cell‐like shape to a relatively elliptical morphology compared to cells treated with TSA alone (Figure 3D). The TSA‐induced alterations in the levels of EMT‐related proteins were at least partially attenuated by U0126 (Figure 3E–3G). These findings demonstrate an essential role of the ERK1/2 pathway, as a critical regulator of Slug, in modulating EMT in TSA‐treated ESCC cells.

### 3.4 TSA promotes ESCC cell migration by activating the ERK1/2‐PAI‐1 pathway

**FIGURE 4:**
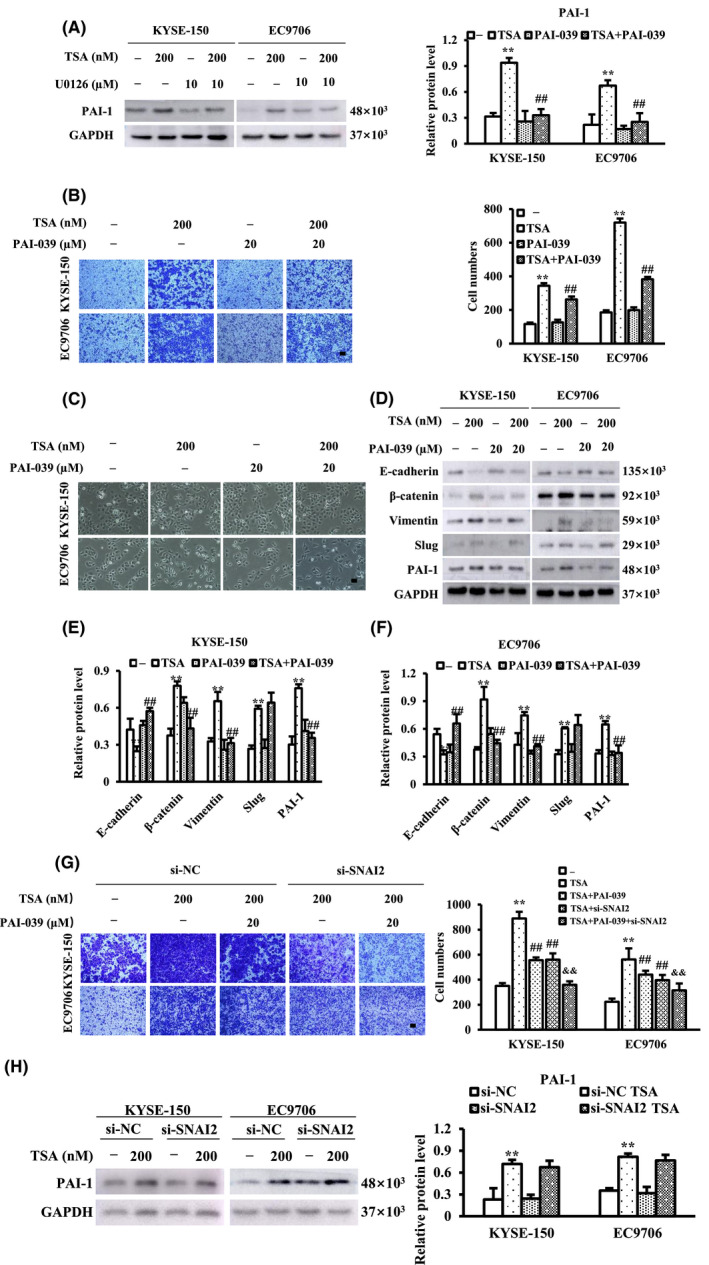
TSA promotes esophageal squamous cell carcinoma (ESCC) cell migration and epithelial‐mesenchymal transition (EMT) by activating ERK/PAI‐1 pathway. (A) ESCC cells were treated with TSA in the absence or presence of U0126 and the PAI‐1 levels were examined by western blot. (B, C and D) ESCC cells were treated with TSA in the absence or presence of PAI‐1 inhibitor PAI‐039, cell migration was studied and migrated cells were quantified (B); images of ESCC cell morphology were taken under the phase contrast Nikon microscope equipped with a digital camera (C) and cell lysates were subjected to immunoblot analysis with the appropriate antibodies (D–F). (G) ESCC cells were treated with TSA in the absence or presence of PAI‐039 and/or si‐SNAI2, cell migration was studied and migrated cells were quantified. (H) The expression of PAI‐1 by western blot after SNAI2 knockdown. Scale bars represents 100 μm, ^**^ *p *< 0.01 versus control. ^##^ *p *< 0.01 versus TSA. ^&&^ *p *< 0.01 versus TSA+PAI‐039 and TSA+si‐SNAI2

Our previous RNA‐sequencing results showed that the expression levels of 73 genes increased more than twofold after TSA treatment (data not shown). Of these 73 genes, plasminogen activator inhibitor 1 (PAI‐1) has attracted particular interest. PAI‐1 has been known for many years as an inhibitor of tissue urokinase plasminogen activator (uPA) and plasminogen activator (tPA), with important roles in regulating cell migration by modulating extracellular matrix proteolysis. Expression of the PAI‐1 gene is promoted by multiple transcription factors, such as activator protein 1 (AP‐1), whose activation is mainly driven by ERK1/2 signaling. Accumulated evidence has shown that ERK1/2‐driven signaling promotes the expression of PAI‐1.^25^ Therefore, we examined the expression of PAI‐1 protein in ESCC cells after TSA treatment in the absence or presence of U0126. TSA evidently increased the expression of PAI‐1 and p‐ERK1/2, whereas U0126 prevented TSA‐induced changes in the expression of these proteins (Figure 4A), indicating that TSA enhances the expression of PAI‐1 through the ERK1/2 signaling pathway in ESCC cells. The effects of PAI‐1 on TSA‐induced ESCC cell migration, morphological changes, and EMT markers were also studied by administrating the PAI‐1 inhibitor PAI‐039. Transwell assays revealed that PAI‐039 suppressed TSA‐induced ESCC cell migration (Figure 4B). PAI‐039 altered the morphology of the majority of TSA‐treated cells from a spindle‐like shape to relatively elliptical or circular in appearance (Figure 4C). Moreover, PAI‐039 at least partially reversed the effects of TSA on protein levels of E‐cadherin, β‐catenin, vimentin, and PAI‐1, but did not influence the expression of Slug (Figure 4D–4F). Combination of Slug and PAI‐1 inhibition revealed stronger ability for inhibiting the migration of ESCC cells compared to alone (Figure 4G). In addition, the knockdown of SNAI2 did not impair the expression of PAI‐1, which was TSA‐induced (Figure 4H). Altogether, these data show that TSA promotes ESCC cell migration by activating ERK1/2‐PAI‐1 signaling, but PAI‐1‐driven EMT in TSA‐induced ESCC cell migration is independent of Slug.

### 3.5 TSA activating ERK pathway may be associated with BRD4

**FIGURE 5:**
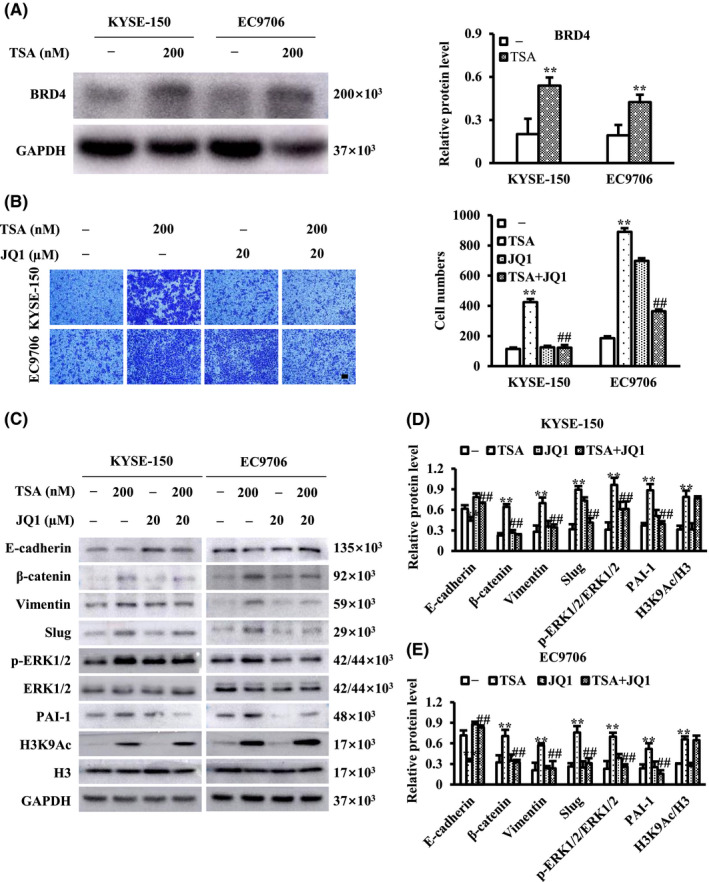
TSA‐induced esophageal squamous cell carcinoma (ESCC) cell migration and epithelial‐mesenchymal transition (EMT) may be associated with BRD4. ESCC cells were treated with TSA in the absence or presence of BRD4 inhibitor JQ1 for 24 h. (A) The expression analysis of BRD4 in ESCC cells by western blot. (B) Cell migration was examined and migrated cells were quantified; (C–E) cell lysates were subjected to immunoblots with the specific antibodies. Scale bar represents 100 μm. ^**^ *p *< 0.01 versus control, ^##^ *p *< 0.01 versus TSA

BRD4 belongs to the bromodomain and extra terminal domain (BET) family and recognizes acetylated lysine residues of histones through two tandem bromodomain modules. BRD4 causes chromatin remodeling and is a major transcriptional regulator. Genome‐wide studies have revealed that class I HDACI‐induced genes were enriched for BRD4 occupancy, indicating a critical role of BRD4 in the transcription of a subgroup of genes regulated by class I HDACI.^26^ In this study, we found that TSA, mainly inhibiting class I and II HDAC, increased the acetylation of H3 in ESCC cells (Figure 1C). Thus, we reasoned that a subgroup of genes induced by TSA would be transactivated in a BRD4‐dependent manner that relies on acetylated H3, resulting in the activation of ERK1/2 in ESCC cells and we found that TSA increased the expression of BRD4 in ESCC cells (Figure 5A). Based on the above, we explored whether BRD4 participates in TSA‐induced activation of ERK1/2 and ESCC cell migration. It was found that JQ1, a BRD4 inhibitor, inhibited TSA‐induced ESCC cell migration (Figure 5B), and at least partially reversed TSA‐induced effects on the levels of p‐ERK1/2, PAI‐1, and all EMT markers examined in this study (Figure 5C–5E). These results suggest that TSA might activate ERK1/2 in a BRD4‐dependent manner, and subsequently promote ESCC cell migration through EMT.

## 4 DISCUSSION

Previous studies have demonstrated that HDACIs, a new class of antitumor drugs, affect a variety of biological processes. These include promoting cell apoptosis and cell cycle arrest and inhibiting EMT, migration, and invasion of cells.^17^, ^27^, ^28^, ^29^ Several HDACIs are in clinical trials for the treatment of hematological malignancies and the HDACI SAHA has been approved by the FDA for the treatment of cutaneous T‐cell lymphoma.^30^ However, recent studies have indicated that HDACIs provide limited benefit to patients with solid tumors and even promote the metastasis of cancer cells.^16^, ^17^, ^18^ TSA, a classic HDACI that inhibits class I and II HDACs has been demonstrated to suppress ESCC cell proliferation,^21^, ^22^ previous study found that TSA promotes ESCC cell migration^23^ and we found that TSA promotes ESCC cell invasion (Figure S1). Thus, combination therapeutic strategies that selectively overcome HDACI‐induced cell migration will provide improved anti‐cancer therapeutic benefits. Here, we describe the possible mechanisms by which TSA promotes ESCC cell migration, supporting the concept of combination therapy with HDACI for ESCC patients.

EMT is a process in which epithelial cells lose cell polarity and intercellular adhesion and transform into a mesenchymal phenotype, thus gaining migratory and invasive traits. EMT contributes to cancer metastatic status and prognosis.^31^, ^32^, ^33^, ^34^ EMT‐related transcription factors, including Snail, Slug, and Twist, potentiate EMT progression by inhibiting the expression of epithelial markers, such as E‐cadherin, and by promoting the expression of mesenchymal markers such as β‐catenin and vimentin.^35^ This study showed that the application of TSA promotes ESCC cell migration and the EMT process. Knockdown of SNAI2 blocked TSA‐induced ESCC cell migration and TSA‐induced upregulation of E‐cadherin and downregulation of β‐catenin and vimentin. In this study, we showed that TSA facilitates ESCC cell migration by promoting slug‐mediated EMT.

ERK1/2 is a proline‐directed serine/threonine protein kinase that participates in diverse cancer processes, including cell migration and invasion. For instance, it has been reported that the ERK1/2‐activated signaling pathway regulates breast cancer cell migration by maintaining Slug expression.^36^ In some cancer cells, TSA has been demonstrated to activate multiple intracellular signaling pathways, such as ERK1/2.^24^ In the present study, we demonstrated that TSA resulted in the activation of ERK1/2 in ESCC cells, whereas inhibition of ERK1/2 activity via application of the MEK1/2 inhibitor U0126 prevented TSA‐induced ESCC migration, EMT, and upregulation of Slug levels, thus suggesting the ERK1/2‐slug pathway as a critical regulator of EMT in TSA‐induced ESCC migration.

Different branches of the ERK1/2 pathways are involved in promoting cell migration.^37^ Our previous RNA‐sequencing results revealed that 73 genes were significantly upregulated twofold in TSA‐treated KYSE‐150 cells when compared with control cells (data not shown). PAI‐1, one of these genes, related to EMT function as well as the ERK1/2 pathway, attracted our attention. PAI‐1 plays important roles in regulating cell adhesion, migration, and invasion.^38^, ^39^, ^40^ It has been reported that PAI‐1 is a poor prognostic factor for disease progression in certain cancer types, such as colon cancer, breast cancer, and glioma.^41^, ^42^, ^43^ In the promoter region of the PAI‐1 gene, there are multiple transcription factor binding sites, such as for the activator protein 1 (AP‐1), whose activation is mainly driven by ERK1/2 signaling. Accumulated evidence also shows that ERK1/2‐driven signaling activates the transcription of PAI‐1.^44^ We demonstrated via RT‐qPCR and western blotting that TSA indeed increased the levels of PAI‐1 mRNA and protein. Interestingly, we found that inhibition of ERK1/2 blocked TSA‐induced increase in PAI‐1 levels in ESCC cells, suggesting that TSA increases PAI‐1 expression via activation of the ERK1/2 signaling pathway. To evaluate the functional consequences of elevated PAI‐1, we applied a PAI‐1 inhibitor and found that the inhibition of PAI‐1 conspicuously prevented TSA‐induced ESCC cell migration, EMT, upregulation of E‐cadherin, and downregulation of β‐catenin and vimentin, but did not inhibit TSA‐induced upregulation of Slug, indicating that the upregulation of PAI‐1 is linked with TSA‐mediated ESCC cell migration via promotion of EMT, which is not related to the alteration of Slug level.

Our data indicate the existence of at least two separable ERK1/2‐dependent signaling pathways in TSA‐mediated ESCC cell migration: an ERK1/2– Slug branch and an ERK1/2‐PAI‐1 branch, and both branches appear to favor the EMT process. ERK1/2 activation is at the core of these two branches. Hence, determination of the mechanism of activation of ERK1/2 is necessary to enhance our understanding of TSA‐regulated signaling systems controlling ESCC cell migration. Increased acetylation levels of histones have been suggested to be associated with HDACI‐based anti‐cancer therapy. BRD4 belongs to the BET family of bromodomain‐containing proteins that function as epigenetic readers of histoneacetyllysine residues that regulate gene transcription. Previous studies have reported that class I HDACI‐induced genes are enriched for BRD4 occupancy, indicating a critical role of BRD4 in the expression of a subgroup of genes induced by class I HDACI.^26^ Recently, Liao et al. have reported that the BRD4 inhibitor JQ1, which binds competitively to the bromodomain and inhibits BRD4 interaction with acetylated histone to suppress the transcription of oncogenes, attenuated ERK1/2 phosphorylation in the NUT midline carcinoma cell line NMC1015.^45^ These findings raise the possibility that BRD4 may play a role in TSA‐driven ERK1/2 activation in ESCC cells. Consistent with the findings of our study, TSA causes an increase in histone H3 acetylation level, while unexpectedly, the BRD4 inhibitor JQ1suppressed TSA‐mediated ESCC cell migration and attenuated TSA‐driven ERK1/2 phosphorylation as well as EMT. Moreover, JQ1 blocked TSA‐induced upregulation in the level of both Slug and PAI‐1. Our data provide mechanistic evidence that BRD4 is responsible for two separable ERK1/2‐dependent signaling pathways in TSA‐mediated ESCC cell migration. Based on previous reports and our findings, it is critical to further identify the molecular machinery that mediates the effect of BRD4 on ERK1/2 activation in TSA‐treated ESCC cells.

## 5 CONCLUSION

**FIGURE 6:**
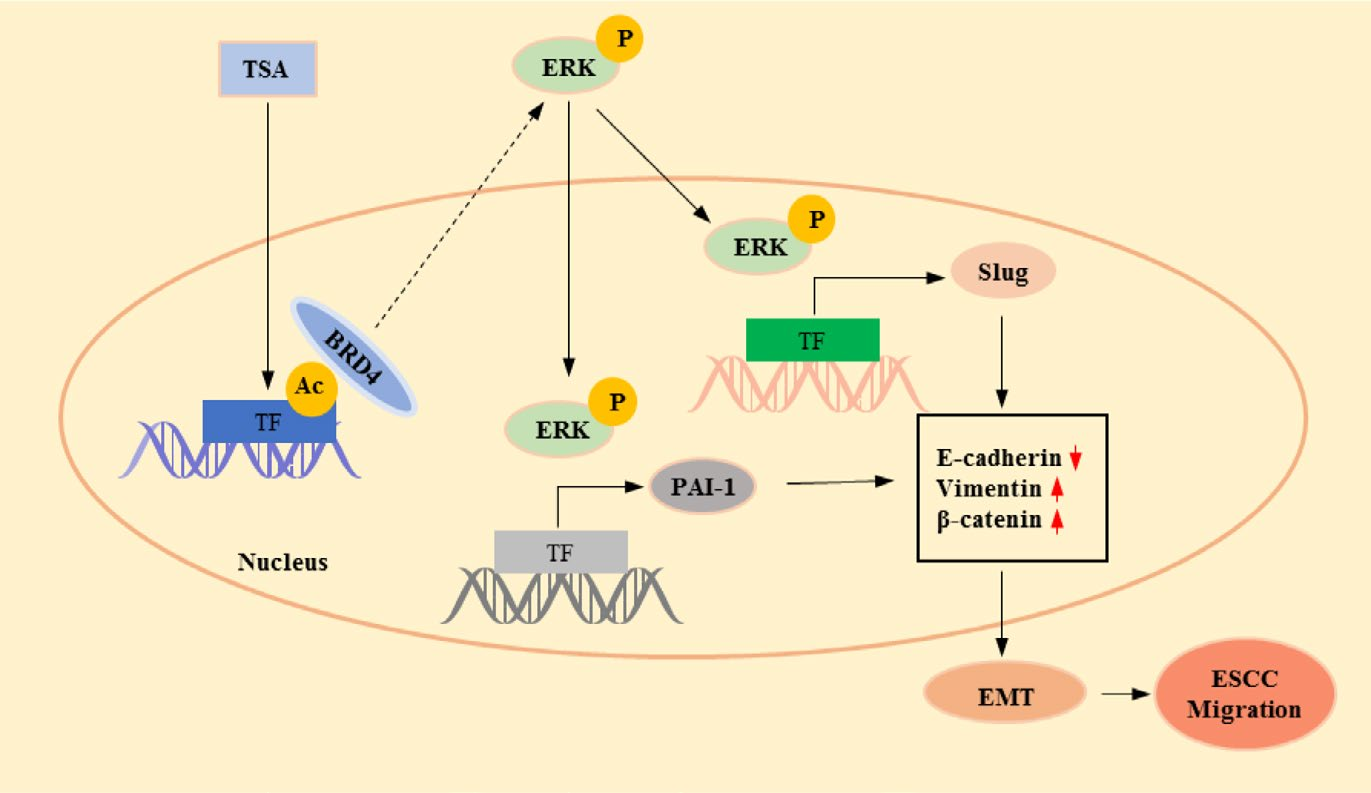
A possible model to illustrate the mechanism of TSA promoting ESCC cell EMT and migration. Ac, acetylation; TF, transcription factor; P, phosphorylation.

We revealed a promising regulatory axis involved in TSA‐mediated ESCC cell migration (Figure 6). Specifically, ERK1/2 activation initiated by BRD4 recruiting acetylated histones or transcription factors plays a central role in TSA‐mediated ESCC cell migration. The two separable ERK1/2‐dependent signaling pathways, ERK1/2‐Slug and ERK1/2‐PAI‐1, contribute to TSA‐mediated ESCC cell migration and EMT. Although further investigation is needed to fully clarify the underlying mechanisms of HDACIs on cancer cell migration, our findings provide the basis for combination therapy with HDACI for ESCC patients to overcome HDACI‐driven cell migration side effects.

## CONFLICT OF INTEREST

The authors declare that they have no known competing financial interests or personal relationships that could have appeared to influence the work reported in this paper.

## ETHICS APPROVAL

Because all information was obtained from public databases, no ethics committee approval is required.

## Supporting information

Fig S1

## Data Availability

All experimental data in this study can be obtained from the corresponding author upon request.

## References

[1] Bray F , Ferlay J , Soerjomataram I , Siegel RL , Torre LA , Jemal A . Global cancer statistics 2018: GLOBOCAN estimates of incidence and mortality worldwide for 36 cancers in 185 countries. CA Cancer J Clin. 2018;68(6):394‐424. 30207593 10.3322/caac.21492

[2] Siegel RL , Miller KD , Jemal A . Cancer statistics, 2019. CA Cancer J Clin. 2019;69(1):7‐34. 30620402 10.3322/caac.21551

[3] Chen W , Zheng R , Baade PD , et al. Cancer statistics in China, 2015. CA Cancer J Clin. 2016;66(2):115‐132. 26808342 10.3322/caac.21338

[4] Cheng J , Jin H , Hou X , Lv J , Gao X , Zheng G . Disturbed tryptophan metabolism correlating to progression and metastasis of esophageal squamous cell carcinoma. Biochem Biophys Res Commun. 2017;486(3):781‐787. 28342863 10.1016/j.bbrc.2017.03.120

[5] Weichert W . HDAC expression and clinical prognosis in human malignancies. Cancer Lett. 2009;280(2):168‐176. 19103471 10.1016/j.canlet.2008.10.047

[6] Montezuma D , Henrique RM , Jerónimo C . Altered expression of histone deacetylases in cancer. Crit Rev Oncog. 2015;20(1‐2):19‐34. 25746102 10.1615/critrevoncog.2014012554

[7] Glozak MA , Seto E . Histone deacetylases and cancer. Oncogene. 2007;26(37):5420‐5432. 17694083 10.1038/sj.onc.1210610

[8] Ma L , Qi L , Li S , et al. Aberrant HDAC3 expression correlates with brain metastasis in breast cancer patients. Thorac Cancer. 2020;11(9):2493‐2505. 32686908 10.1111/1759-7714.13561 PMC7471029

[9] Eto S , Saeki K , Yoshitake R , et al. Anti‐tumor effects of the histone deacetylase inhibitor vorinostat on canine urothelial carcinoma cells. PLoS One. 2019;14(6):e0218382. 31206526 10.1371/journal.pone.0218382 PMC6576781

[10] Weinlander E , Somnay Y , Harrison AD , et al. The novel histone deacetylase inhibitor thailandepsin A inhibits anaplastic thyroid cancer growth. J Surg Res. 2014;190(1):191‐197. 24679699 10.1016/j.jss.2014.02.042 PMC4063213

[11] Wang L , Bao X , Yang J , et al. Novel cinnamohydroxamic acid derivatives as HDAC inhibitors with anticancer activity in vitro and in vivo. Chem Biol Interact. 2016;249:64‐70. 26944433 10.1016/j.cbi.2016.02.018

[12] Srinivas C , Swathi V , Priyanka C , et al. Novel SAHA analogues inhibit HDACs, induce apoptosis and modulate the expression of microRNAs in hepatocellular carcinoma. Apoptosis. 2016;21(11):1249‐1264. 27502208 10.1007/s10495-016-1278-6

[13] Lee SY , Hong EH , Jeong JY , et al. Esterase‐sensitive cleavable histone deacetylase inhibitor‐coupled hyaluronic acid nanoparticles for boosting anticancer activities against lung adenocarcinoma. Biomater Sci. 2019;7(11):4624‐4635. 31451819 10.1039/c9bm00895k

[14] Bertino EM , Otterson GA . Romidepsin: a novel histone deacetylase inhibitor for cancer. Expert Opin Investig Drugs. 2011;20(8):1151‐1158. 10.1517/13543784.2011.594437 21699444

[15] Piekarz RL , Frye R , Prince HM , et al. Phase 2 trial of romidepsin in patients with peripheral T‐cell lymphoma. Blood. 2011;117(22):5827‐5834. 21355097 10.1182/blood-2010-10-312603 PMC3112033

[16] Lin K‐T , Wang Y‐W , Chen C‐T , Ho C‐M , Su W‐H , Jou Y‐S . HDAC inhibitors augmented cell migration and metastasis through induction of PKCs leading to identification of low toxicity modalities for combination cancer therapy. Clin Cancer Res. 2012;18(17):4691‐4701. 22811583 10.1158/1078-0432.CCR-12-0633

[17] Ji M , Lee EJ , Kim KB , et al. HDAC inhibitors induce epithelial‐mesenchymal transition in colon carcinoma cells. Oncol Rep. 2015;33(5):2299‐2308. 25813246 10.3892/or.2015.3879

[18] Wang J , Xu M‐Q , Jiang X‐L , Mei X‐Y , Liu X‐G . Histone deacetylase inhibitor SAHA‐induced epithelial‐mesenchymal transition by upregulating Slug in lung cancer cells. Anticancer Drugs. 2018;29(1):80‐88. 29176396 10.1097/CAD.0000000000000573

[19] Toh Y , Yamamoto M , Endo K , et al. Histone H4 acetylation and histone deacetylase 1 expression in esophageal squamous cell carcinoma. Oncol Rep. 2003;10(2):333‐338. 12579268

[20] Wang F , Qi Y , Li X , He W , Fan Q‐X , Zong H . HDAC inhibitor trichostatin A suppresses esophageal squamous cell carcinoma metastasis through HADC2 reduced MMP‐2/9. Clin Invest Med. 2013;36(2):E87‐E94. 23544610 10.25011/cim.v36i2.19571

[21] Ma J , Guo X , Zhang S , et al. Trichostatin A, a histone deacetylase inhibitor, suppresses proliferation and promotes apoptosis of esophageal squamous cell lines. Mol Med Rep. 2015;11(6):4525‐4531. 25634603 10.3892/mmr.2015.3268

[22] Cheng YW , Liao LD , Yang Q , et al. The histone deacetylase inhibitor panobinostat exerts anticancer effects on esophageal squamous cell carcinoma cells by inducing cell cycle arrest. Cell Biochem Funct. 2018;36(8):398‐407. 30484863 10.1002/cbf.3359

[23] Huang K . Trichostatin A augments esophageal squamous cell carcinoma cells migration by inducing acetylation of RelA at K310 leading epithelial‐mesenchymal transition. Anticancer Drugs. 2020;31(6):567‐574. 32282366 10.1097/CAD.0000000000000927

[24] Yao J , Qian C‐J , Ye B , Zhang X , Liang Y . ERK inhibition enhances TSA‐induced gastric cancer cell apoptosis via NF‐κB‐dependent and Notch‐independent mechanism. Life Sci. 2012;91(5‐6):186‐193. 22781708 10.1016/j.lfs.2012.06.034

[25] Talati N , Kamato D , Piva TJ , Little PJ , Osman N . Thrombin promotes PAI‐1 expression and migration in keratinocytes via ERK dependent Smad linker region phosphorylation. Cell Signal. 2018;47:37‐43. 29577978 10.1016/j.cellsig.2018.03.009

[26] Mishra VK , Wegwitz F , Kosinsky RL , et al. Histone deacetylase class‐I inhibition promotes epithelial gene expression in pancreatic cancer cells in a BRD4‐and MYC‐dependent manner. Nucleic Acids Res. 2017;45(11):6334‐6349. 28369619 10.1093/nar/gkx212 PMC5499659

[27] Song X , Wu JQ , Yu XF , Yang XS , Yang Y . Trichostatin A inhibits proliferation of triple negative breast cancer cells by inducing cell cycle arrest and apoptosis. Neoplasma. 2018;65(6):898‐906. 30334455 10.4149/neo_2018_181212N476

[28] Bruzzese F , Leone A , Rocco M , et al. HDAC inhibitor vorinostat enhances the antitumor effect of gefitinib in squamous cell carcinoma of head and neck by modulating ErbB receptor expression and reverting EMT. J Cell Physiol. 2011;226(9):2378‐2390. 21660961 10.1002/jcp.22574

[29] Bian X , Liang Z , Feng A , Salgado E , Shim H . HDAC inhibitor suppresses proliferation and invasion of breast cancer cells through regulation of mir‐200c targeting CRKL. Biochem Pharmacol. 2018;147:30‐37. 29155146 10.1016/j.bcp.2017.11.008 PMC5733635

[30] Duvic M , Talpur R , Ni X , et al. Frankel SR (2007) Phase 2 trial of oral vorinostat (suberoylanilide hydroxamic acid, SAHA) for refractory cutaneous T‐cell lymphoma (CTCL). Blood. 2007;109(1):31‐39. 16960145 10.1182/blood-2006-06-025999 PMC1785068

[31] Zeisberg EM , Tarnavski O , Zeisberg M , et al. Endothelial‐to‐mesenchymal transition contributes to cardiac fibrosis. Nat Med. 2007;13(8):952‐961. 17660828 10.1038/nm1613

[32] Aiello NM , Kang Y . Context‐dependent EMT programs in cancer metastasis. J Exp Med. 2019;216(5):1016‐1102. 30975895 10.1084/jem.20181827 PMC6504222

[33] Bischoff J . Endothelial‐to‐Mesenchymal Transition. Circ Res. 2019;124(8):1163‐1165. 30973806 10.1161/CIRCRESAHA.119.314813 PMC6540806

[34] Li H , Zhang J , Song X , et al. Alveolar epithelial cells undergo epithelial‐mesenchymal transition in acute interstitial pneumonia: a case report. BMC Pulm Med. 2014;14:67‐73. 24755111 10.1186/1471-2466-14-67 PMC4013083

[35] Xin Y , Cummins B , Gedeon T . Multistability in the epithelial‐mesenchymal transition network. BMC Bioinformatics. 2020;21(1):71‐88. 32093616 10.1186/s12859-020-3413-1 PMC7041120

[36] Chen H , Zhu G , Li Y , et al. Extracellular signal‐regulated kinase signaling pathway regulates breast cancer cell migration by maintaining slug expression. Cancer Res. 2009;69(24):9228‐9235. 19920183 10.1158/0008-5472.CAN-09-1950 PMC2795125

[37] Hirata E , Kiyokawa E . ERK activity imaging during migration of living cells in vitro and in vivo. Int J Mol Sci. 2019;20(3):679‐695. 10.3390/ijms20030679 PMC6387119 30764494

[38] Tang L , Han X . The urokinase plasminogen activator system in breast cancer invasion and metastasis. Biomed Pharmacother. 2013;67(2):179‐182. 23201006 10.1016/j.biopha.2012.10.003

[39] Thapa B , Kim YH , Kwon HJ , et al. The LRP1‐independent mechanism of PAI‐1‐induced migration in CpG‐ODN activated macrophages. Int J Biochem Cell Biol. 2014;49:17‐25. 24440681 10.1016/j.biocel.2014.01.008

[40] Li S , Wei X , He J , et al. Plasminogen activator inhibitor‐1 in cancer research. Biomed Pharmacother. 2018;105:83‐94. 29852393 10.1016/j.biopha.2018.05.119

[41] Hogan NM , Joyce MR , Murphy JM , et al. Impact of mesenchymal stem cell secreted PAI‐1 on colon cancer cell migration and proliferation. Biochem Biophys Res Commun. 2013;435(4):574‐579. 23685140 10.1016/j.bbrc.2013.05.013

[42] Wei X , Li S , He J , et al. Tumor‐secreted PAI‐1 promotes breast cancer metastasis via the induction of adipocyte‐derived collagen remodeling. Send to Cell Commun Signal. 2019;17(1):58‐75. 10.1186/s12964-019-0373-z PMC6554964 31170987

[43] Hlavaty J , Ertl R , Miller I , Gabriel C . Expression of progesterone receptor membrane component 1 (PGRMC1), progestin and AdipoQ receptor 7 (PAQPR7), and plasminogen activator inhibitor 1 RNA‐binding protein (PAIRBP1) in glioma spheroids in vitro. Biomed Res Int. 2016;2016:8065830. 27340667 10.1155/2016/8065830 PMC4908248

[44] Shyu HW , Lin YY , Chen LC , et al. The dengue virus envelope protein induced PAI‐1 gene expression via MEK/ERK pathways. Thromb Haemost. 2010;104(6):1219‐1227. 20886187 10.1160/TH10-05-0302

[45] Liao S , Maertens O , Cichowski K , Elledge SJ . Genetic modifiers of the BRD4‐NUT dependency of NUT midline carcinoma uncovers a synergism between BETis and CDK4/6is. Genes Dev. 2018;32(17‐18):1188‐1200. 30135075 10.1101/gad.315648.118 PMC6120715

