# Supplementary material for: Trichostatin A promotes esophageal squamous cell carcinoma cell migration and EMT through BRD4/ERK1/2‐dependent pathway
Source: Cancer Med. 2021 Jun 23;10(15):5235–45. doi: 10.1002/cam4.4059 (PMC8335841; doi:10.1002/cam4.4059)
Supplement: Supplementary file 1 — Fig S1 [file CAM4-10-5235-s001.pdf]

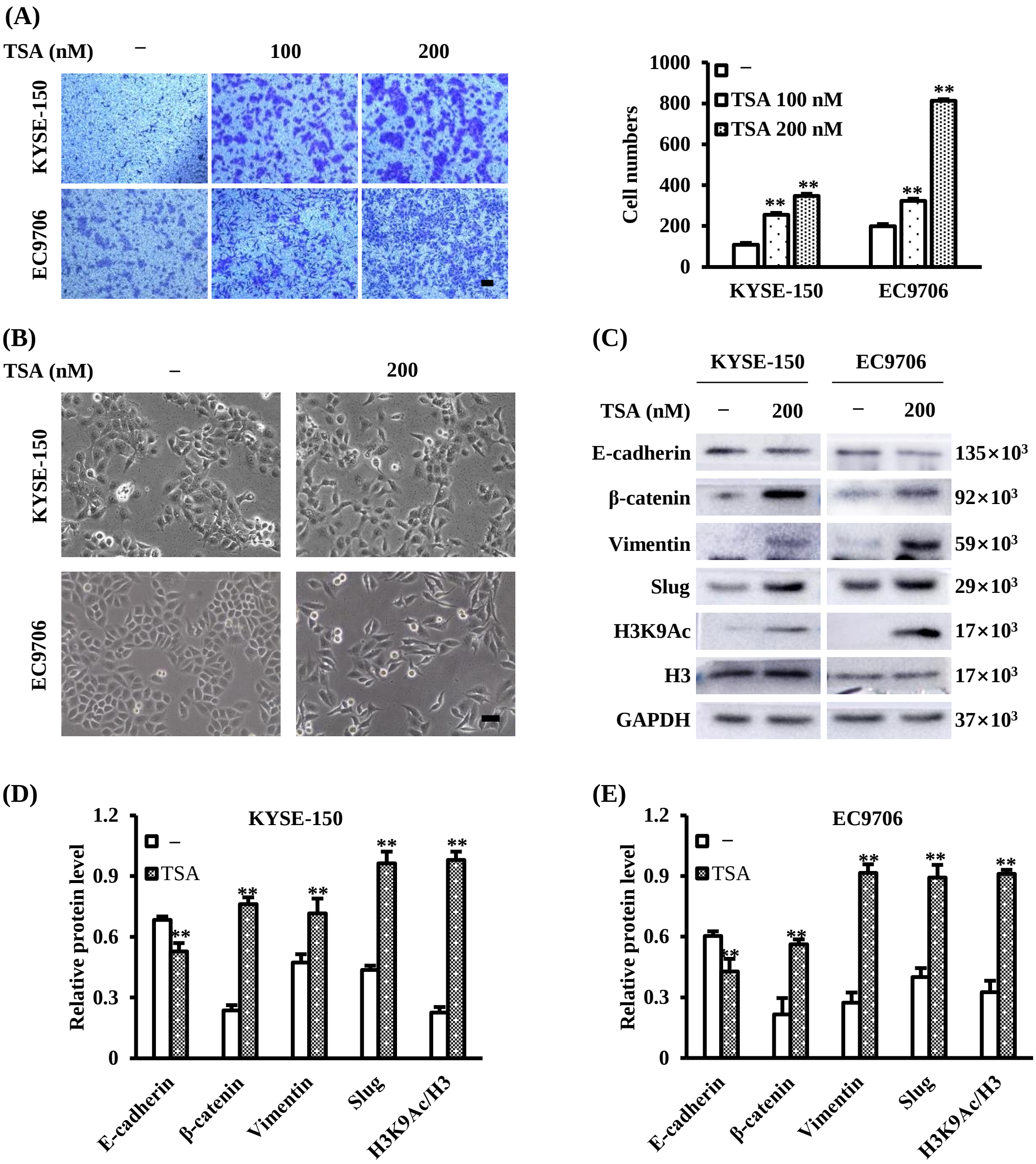

F I G U R E 1 TSA promotes esophageal squamous cell carcinoma (ESCC) cell migration and epithelial-mesenchymal transition (EMT). (A) TSA-induced ESCC cell migration was assessed by transwell assay. (B) Phase contrast images of ESCC cells treated with TSA were captured by a Nikon digital microscope. (C-E) Protein expression levels of EMT markers and H3K9Ac were analyzed by western blot. $**p<0.01$ vs. control. Scale bars in (A) and (B) are 100 μm.

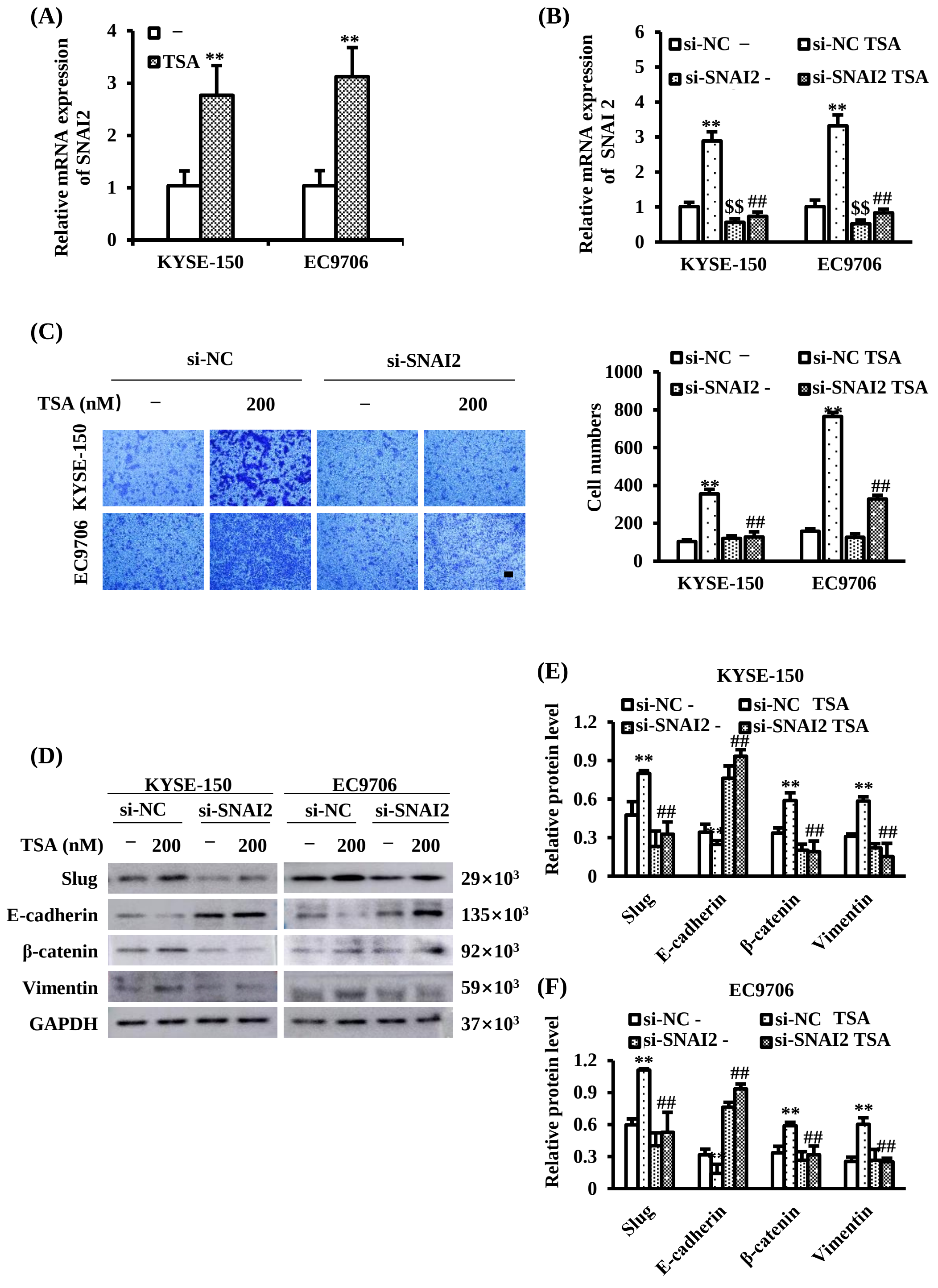
(A)
Relative mRNA expression of SNAI2
TSA
KYSE-150
EC9706
(B)
Relative mRNA expression of SNAI 2
si-NC –
si-NC TSA
si-SNAI2 -
si-SNAI2 TSA
KYSE-150
EC9706
(C)
si-NC
si-SNAI2
TSA (nM)
200
KYSE-150
EC9706
Cell numbers
(D)
Slug
E-cadherin
β-catenin
Vimentin
GAPDH
29×10³
135×10³
92×10³
59×10³
37×10³
(E)
KYSE-150
Relative protein level
(F)
EC9706

F I G U R E 2 TSA facilitates migration of esophageal squamous cell carcinoma (ESCC) cells by increasing the expression of Slug. (A) Relative levels of SNAI2 mRNA in ESCC cells after treatment with TSA was examined by RT-qPCR. (B) Relative level of SNAI2 mRNA in control cells and SNAI2-knockdown cells treated with TSA was examined by RT-qPCR. (C) Cell migration of control cells and SNAI2-knockdown cells was examined by transwell assay, and migrated cells were quantified. (D-F) The expression levels of epithelial-mesenchymal transition (EMT) markers in control cells and SNAI2-knockdown cells after treatment with TSA. Scale bar represents 100 μm. $^{**}p<0.01$ vs. control, $^{\$\$}p<0.01$ vs. control, $^{\#\#}p<0.01$ vs. TSA.

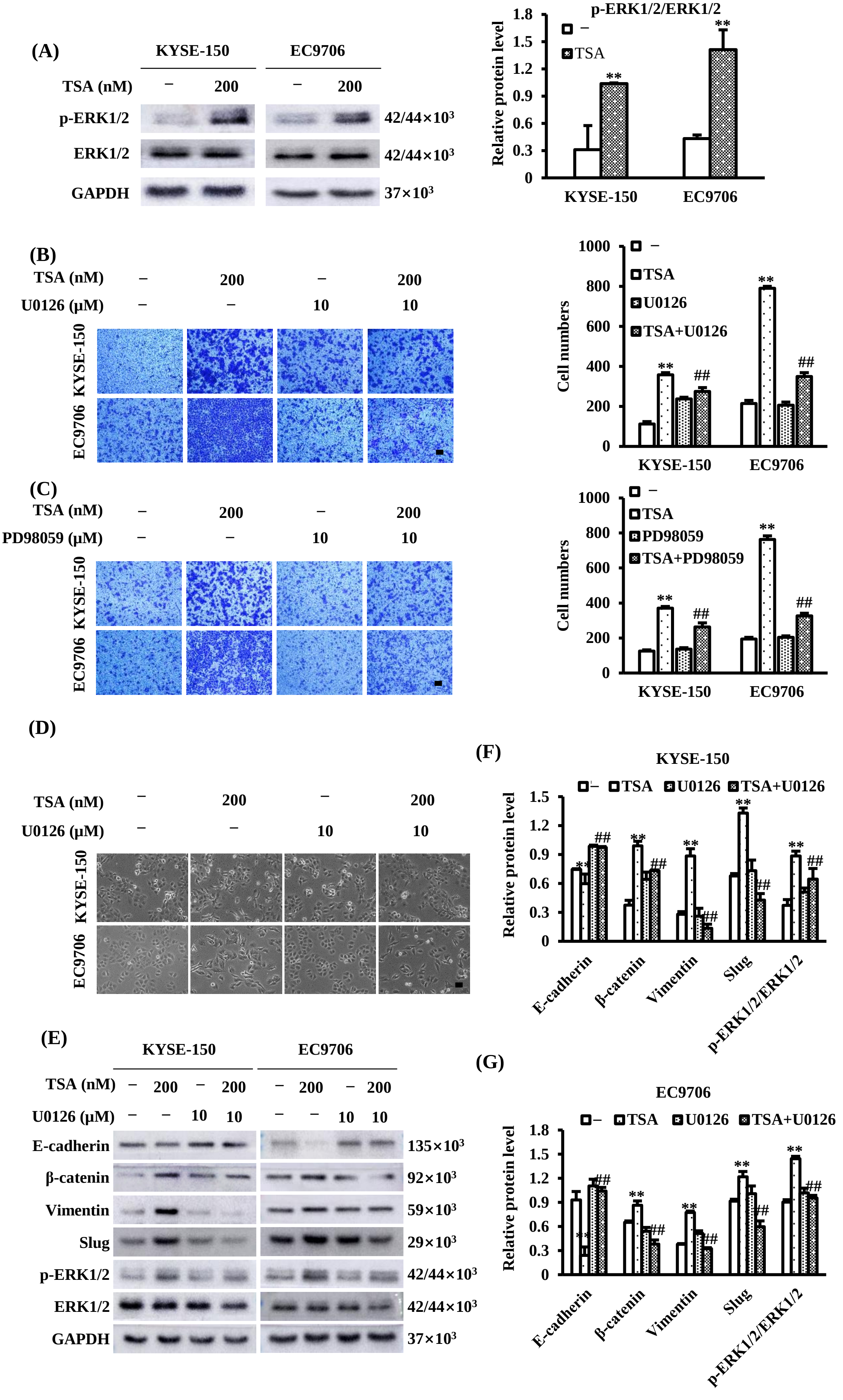

(A)
KYSE-150
EC9706
TSA (nM)
–
200
p-ERK1/2
ERK1/2
GAPDH
42/44×10³
37×10³
p-ERK1/2/ERK1/2
Relative protein level
1.8
1.5
1.2
0.9
0.6
0.3
0
TSA
**
(B)
U0126 (μM)
10
Cell numbers
1000
800
600
400
200
U0126
TSA+U0126
##
(C)
PD98059 (μM)
PD98059
TSA+PD98059
(D)
(E)
E-cadherin
β-catenin
Vimentin
Slug
135×10³
92×10³
59×10³
29×10³
(F)
p-ERK1/2/ERK1/2
(G)

F I G U R E 3 TSA promotes esophageal squamous cell carcinoma (ESCC) cell migration and epithelial-mesenchymal transition (EMT) by activating ERK1/2 pathway. (A)Phosphorylation of ERK1/2 (p-ERK1/2) after treatment of ESCC cells with TSA for 24 h was examined by western blot. (B and C) ESCC cells were treated with TSA in the absence or presence of U0126 and PD98059 for 24 h. Cell migration was measured and migrated cells were quantified. (D and E) ESCC cells were treated with 200 nM TSA in the absence or presence of 10 μM U0126. The cell morphology was captured by a phase contrast Nikon microscope equipped with a digital camera (D) and cell lysates were subjected to immunoblot analysis with the appropriate antibodies (E-G). Scale bars represents 100 μm. $^{**}p$<0.01 vs. control. $^{\#\#}p$<0.01 vs. TSA.

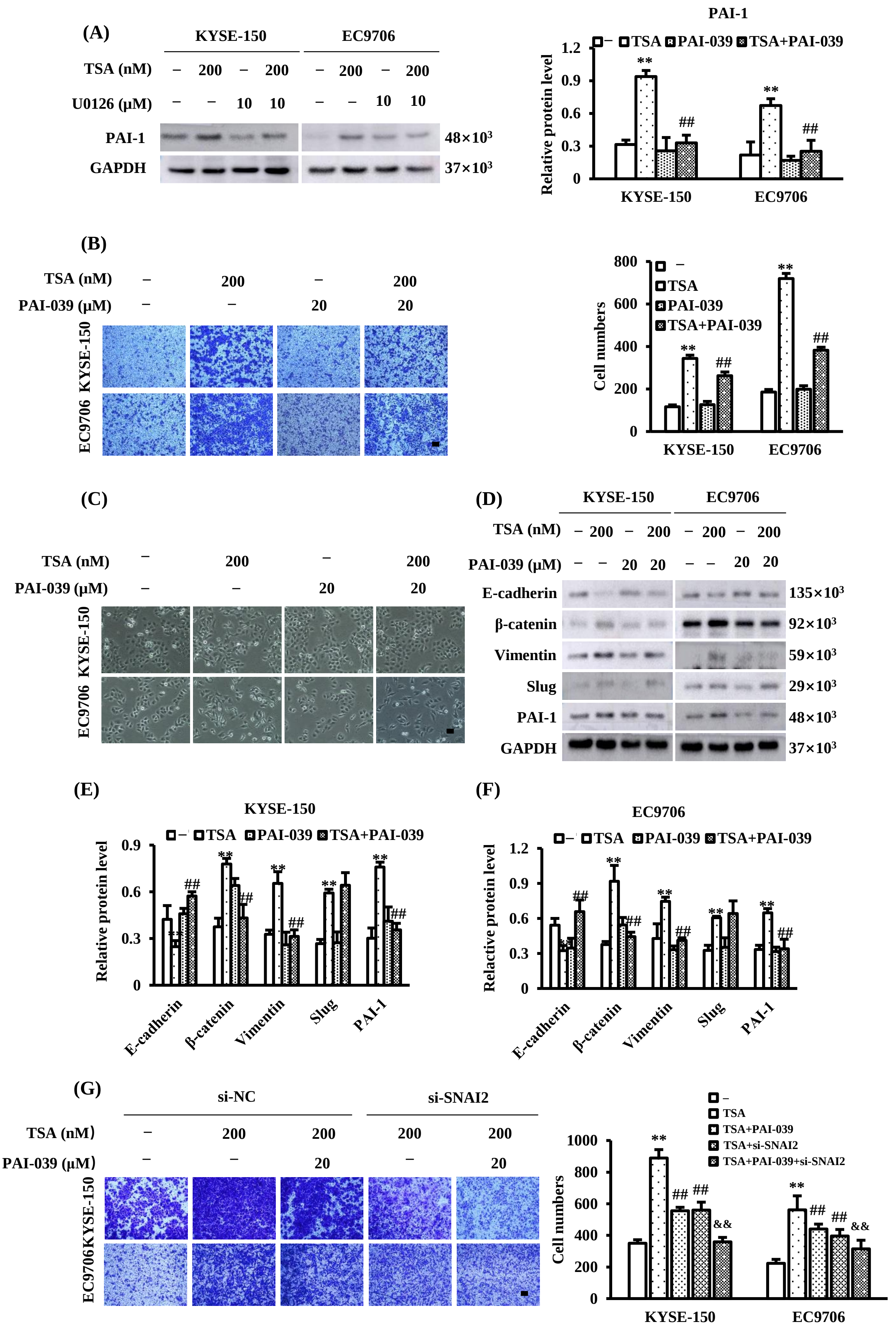

(A)
KYSE-150
EC9706
TSA (nM)
U0126 (μM)
PAI-1
GAPDH
48×10³
37×10³
PAI-1
Relative protein level
– TSA PAI-039 TSA+PAI-039
(B)
TSA (nM)
PAI-039 (μM)
Cell numbers
(C)
(D)
E-cadherin
β-catenin
Vimentin
Slug
135×10³
92×10³
59×10³
29×10³
(E)
(F)
Relactive protein level
(G)
si-NC
si-SNAI2
TSA+si-SNAI2
TSA+PAI-039+si-SNAI2

(H)

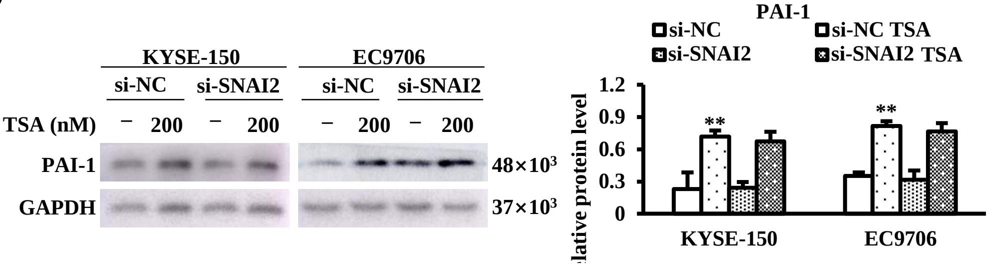

F I G U R E 4 TSA promotes esophageal squamous cell carcinoma (ESCC) cell migration and epithelial-mesenchymal transition (EMT) by activating ERK/PAI-1 pathway. (A) ESCC cells were treated with TSA in the absence or presence of U0126 and the PAI-1 levels were examined by western blot.(B, C and D) ESCC cells were treated with TSA in the absence or presence of PAI-1 inhibitor PAI-039, cell migration was studied and migrated cells were quantified (B); images of ESCC cell morphology were taken under the phase contrast Nikon microscope equipped with a digital camera (C) and cell lysates were subjected to immunoblot analysis with the appropriate antibodies (D-F). (G) ESCC cells were treated with TSA in the absence or presence of PAI-039 and/or si-SNAI2, cell migration was studied and migrated cells were quantified. (H) The expression of PAI-1 by western blot after SNAI2 knockdown. Scale bars represents 100 μm, $^{**}p<0.01$ vs. control. $^{\#\#}p<0.01$ vs. TSA. $^{\&\&}p<0.01$ vs. TSA+PAI-039 and TSA+si-SNAI2.

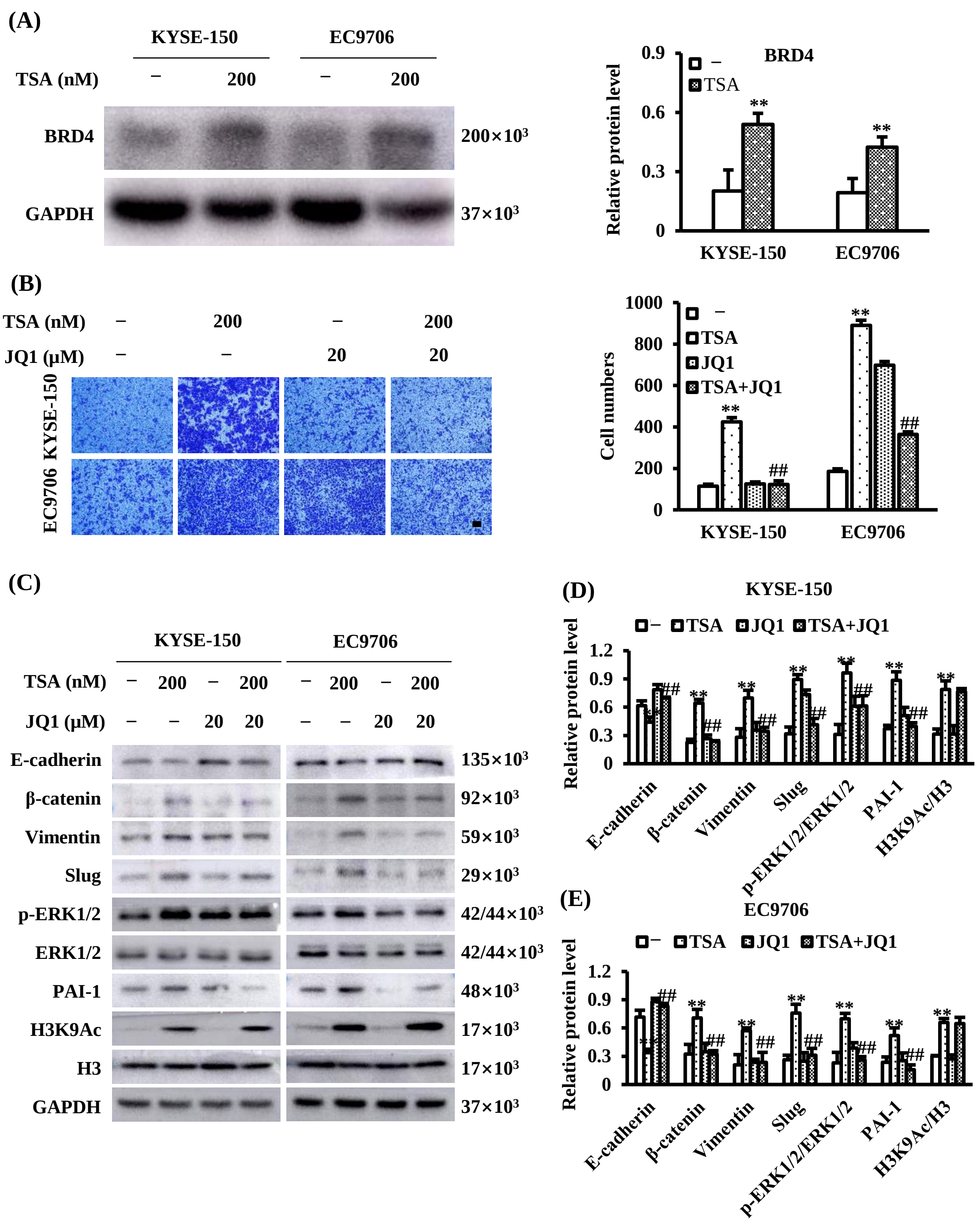

F I G U R E 5 TSA-induced esophageal squamous cell carcinoma (ESCC) cell migration and epithelial-mesenchymal transition (EMT) may be associated with BRD4. ESCC cells were treated with TSA in the absence or presence of BRD4 inhibitor JQ1 for 24 h. (A) The expression analysis of BRD4 in ESCC cells by western blot. (B) Cell migration was examined and migrated cells were quantified; (C-E) cell lysates were subjected to immunoblots with the specific antibodies. Scale bar represents 100 μm. $^{**}p$<0.01 vs. control, $^{\#\#}p$<0.01 vs. TSA.

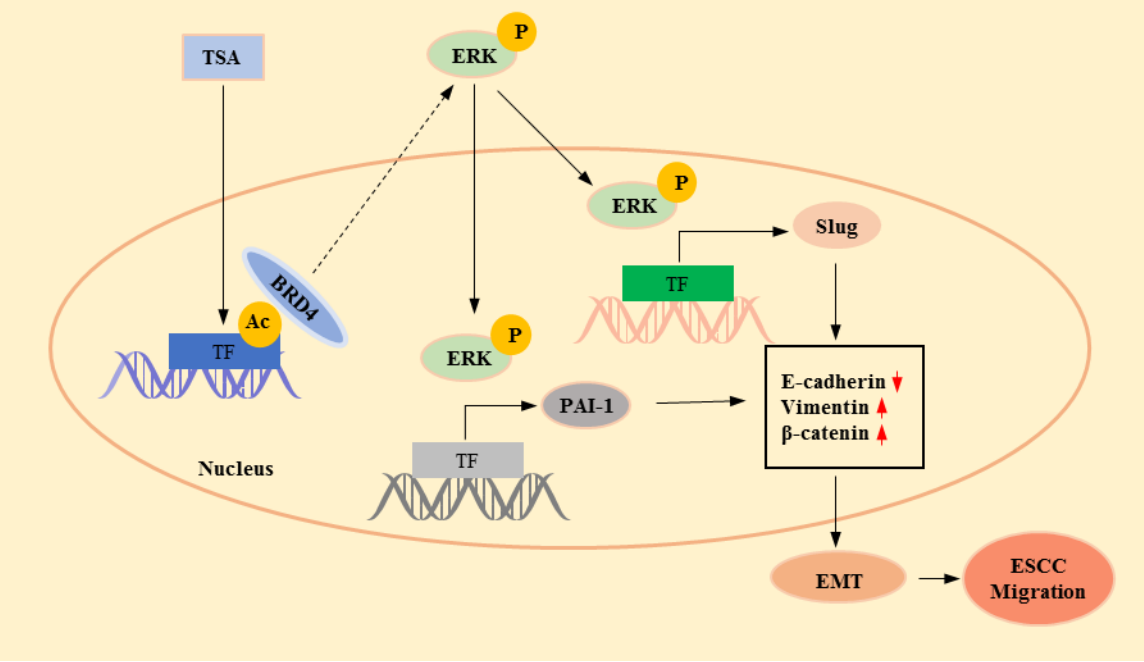

F I G U R E 6 A possible model to illustrate the mechanism of TSA promoting ESCC cell EMT and migration. Ac: acetylation; TF: transcription factor; P: phosphorylation.

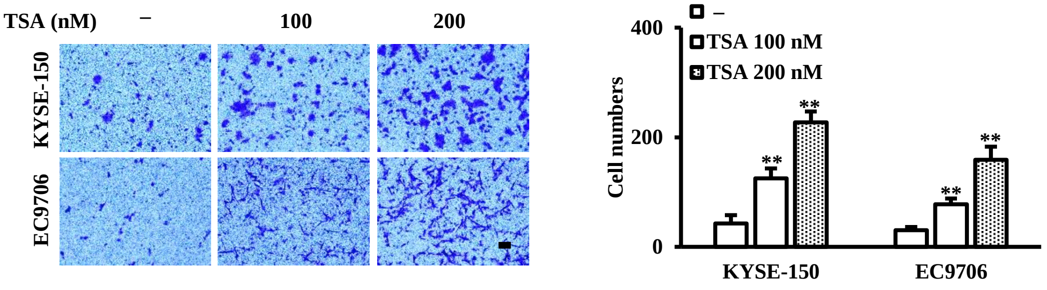

F I G U R E S1 TSA promotes ESCC cells invasion. TSA-mediated ESCC cells invasion was assessed by transwell invasion assay, **p<0.01 vs control. Scale bars are 100 μm.
